# Arthrogryposis multiplex congenita in a child with congenital fractures: a case report

**DOI:** 10.1186/s13256-022-03587-1

**Published:** 2022-10-19

**Authors:** Kavinda Dayasiri, Heshan Jayaweera

**Affiliations:** 1 Department of Paediatrics, Faculty of Medicine, University of Kelaniya, Colombo, Sri Lanka; 2 Department of Paediatrics, University of Peradeniya, Peradeniya, Sri Lanka

**Keywords:** Arthrogryposis, Bruck syndrome, Case report

## Abstract

**Background:**

Bruck syndrome is an exceedingly rare form of osteogenesis imperfecta, inherited autosomal recessively and presenting with the concurrence of bone fragility and congenital contractures of large joints. The disease usually progresses relentlessly to result in recurrent fractures, short stature, severe kyphoscoliosis, and susceptibility to recurrent respiratory tract infections.

**Case presentation:**

The index child was a male newborn to healthy, nonconsanguineous, Sinhalese parents. The child had multiple contractures involving all large joints with pterigium formation in addition to congenital fractures involving left humerus and ulna at birth. The phenotypic features in this child were highly suggestive of Bruck syndrome. Genetic counseling was offered to the parents, although specific genetic testing could not be undertaken due to lack of resources. Bone and skin biopsy were not performed since only palliative care was possible. Over the course, he developed recurrent severe chest infections due to poor muscle tone, weak cough reflex, and pooling of secretions. Unfortunately, he succumbed at the age of 7 months following severe pneumonia.

**Conclusion:**

The association of arthrogryposis with osteogenesis imperfecta is extremely rare and known as Bruck syndrome. Early diagnosis during the antenatal period is helpful in genetic counseling, assessment of severity, and exploration of therapeutic options

## Background

Arthrogryposis multiplex congenita (AMC) is a symptom complex rather than a diagnosis and associated with many recognizable syndromes [1]. Chromosomal abnormalities, uterine abnormalities, and disorders of nerves, vessels, muscles, and connective tissues can lead to this symptom complex. AMC presents with congenital joint contractures. Bruck syndrome is identified when congenital joint contractures are associated with bone fragility and fractures [2]. It has been considered as an autosomal recessive form of osteogenesis imperfecta [3]. This rare disorder has been reported in literature in only 27 patients by 2015 [4]. Viljoen *et al.* reported five children with congenital joint contractures with fractures in 1989 [5]. Since the same features were described in 1897 by Dr. Bruck [6], Viljoen named the condition as Bruck syndrome. The genotype and phenotype of Bruck syndrome are heterogeneous. While the genes coding for collagen 1 chains are unaffected in BS, there is biochemical evidence for a defect in the hydroxylation of lysine residues in collagen 1 telopeptides [7]. We reported herein the case of a neonate with congenital joint contractures associated with bone fragility and fractures and meeting clinical criteria for Bruck syndrome [4].

## Case report

A male newborn to healthy, nonconsanguineous Sinhalese parents at 37 weeks of gestation was identified to have multiple congenital abnormalities. The mother noted reduced fetal movements during pregnancy. Breech presentation and placenta previa were confirmed by antenatal ultrasound with no evidence of oligo- or polyhydramnios. The antenatal period had been otherwise uncomplicated, and anomaly ultrasound scan did not reveal specific congenital abnormalities. He was delivered by elective lower segment cesarian section, weighing 2.6 kg, at Peradeniya Teaching Hospital, Peradeniya, Sri Lanka. Length and occipitofrontal circumference were 48 cm and 34 cm, respectively. Apgar scores were 8, 9, and 10 at 1, 5, and 10 minutes. Vital parameters soon after birth were heart rate of 114 per minute, respiratory rate of 52 per minute, and oxygen saturation of 94% in room air.

Physical examination of the newborn revealed facial asymmetry and micrognathia. Neck muscles were thin and atrophied. Contractures involving all large joints were noticed with pterigium formation. Hands and feet were deformed with absent skin creases. Right talipes equinovarus and left calcaneovarus deformity with dislocated hip joints were also noted. The baby had microphallus with bilateral cryptorchidism. Based on these clinical findings, AMC was considered. Additionally, the child was found to have a disproportionately large head, disproportionately short limbs, scoliosis, and multiple fractures involving left humerus and ulna. Cardiovascular, respiratory, neurological, and abdominal examinations findings were otherwise normal at birth. Serum calcium, phosphate, alkaline phosphatase, and vitamin D levels were within normal ranges. X-ray of left arm and forearm revealed multiple fractures involving humerus and ulna. Hearing and visual assessments revealed normal findings. Ultrasound abdomen did not reveal any congenital abnormalities of the visceral organs.

Bone and skin biopsy were not performed since only palliative care was possible. Similarly, genetic studies for Bruck syndrome were not done due to limited resources. Establishing of feeding and handling was difficult due to increased fragility of bones and evidence of multiple congenital contractures. The baby was treated with vitamin D, calcium, and bisphosphonates and followed up by the pediatric and orthopedic teams.

**Fig. 1:**
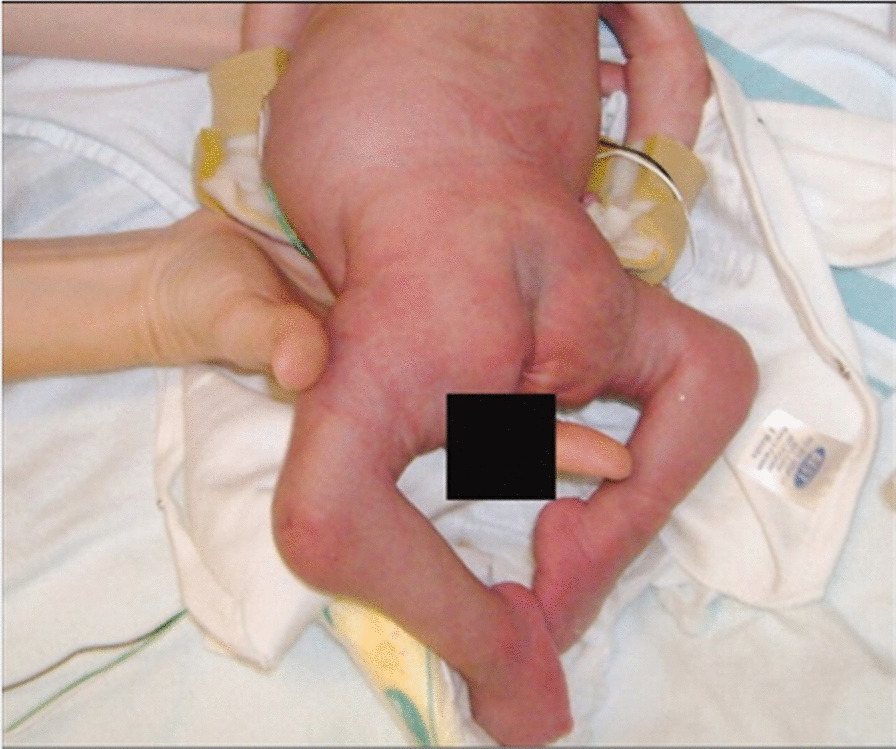
Multiple congenital contractures

**Fig. 2:**
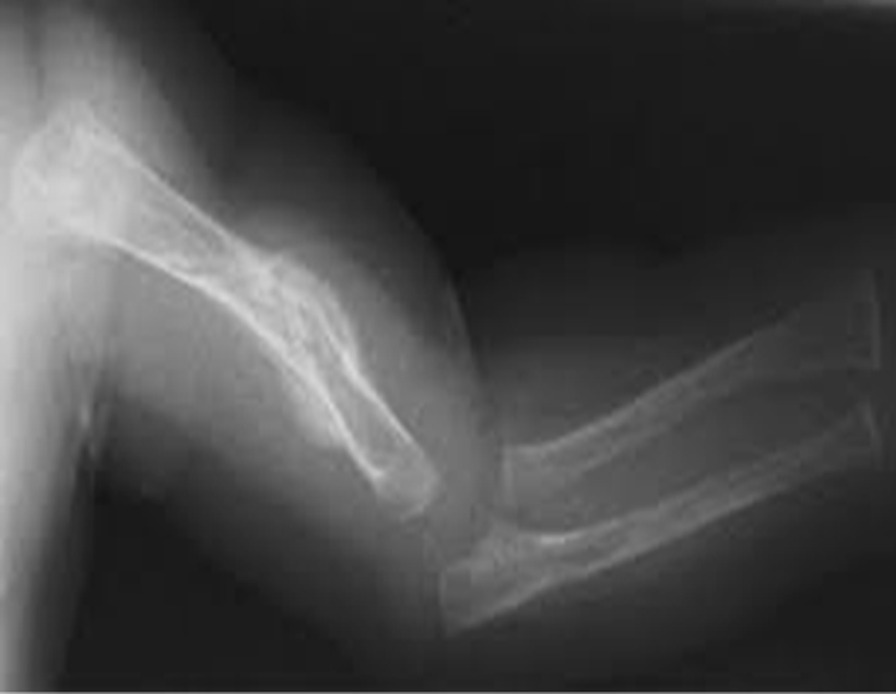
Healing intrauterine fractures

He had recurrent chest infections since birth, managed in-patient with intravenous broad-spectrum antibiotics and supportive care. He had a severe chest infection at the age of 5 months, needing management in the intensive care unit. He was intubated and provided with ventilatory care for 3 days. Intravenous cefotaxime was given for 10 days, and oral clarithromycin for 5 days. The chest infections were largely attributed to weak cough reflex, pooling of secretions, and poor muscle tone, including that of respiration muscles. He remained bed-ridden as he had severe and predominant gross motor developmental delay, which was further complicated by presence of multiple long-bone fractures. He received chest physiotherapy and gentle limb physiotherapy by the multidisciplinary team. Unfortunately, the child succumbed due to respiratory failure following severe pneumonia at age 7 months (Figs. 1, 2).

The second child born to the same parents also had clinical features suggestive of Bruck syndrome. He is currently 6 years of age and doing well with multidisciplinary follow-up. Further, he has normal intellectual functions. He is able to use his hands reasonably well in writing. The main concern of the parents is his limited mobility, and the parents carry him from place to place but his weight is becoming an issue. His current weight is 23 kg. He is toilet trained and suffers from infrequent chest infections, which are managed aggressively. However, he has not been hospitalized, except for once during the last 3 years. Perhaps COVID-19 had a positive effect as his younger sibling was not schooling, thus cross-infections were low. The parents are exploring the possibility of getting a wheelchair for him.

## Discussion

Bruck syndrome is characterized by congenital contractures with pterygia, onset of fractures in infancy or early childhood, severe limb deformity, and progressive scoliosis [8]. It has been classified into two forms—Bruck syndrome type 1 and type 2; however, both variants share common phenotypic features [9]. Bruck syndrome 2 is caused by homozygous mutation in the *PLOD2* gene on chromosome 3q24. The syndrome has also been reported secondary to insertion/deletion mutation in the *FKBP10* gene [10, 11]. Phenotypic features such as short stature and progressive kyphoscoliosis have been suggested to be more associated with Bruck syndrome type 2 [12]. However, given that the index child succumbed early following a severe respiratory tract infection and respiratory failure, determination of the type of Bruck syndrome based on phenotypic features was difficult.

Bruck syndrome is inherited in an autosomal recessive manner. Although there was no previous family history, presence of similar clinical features in both children supports the already known pattern of inheritance in this family. Consanguinity can be absent in some families [13]. Many centers in developing countries, at present, do not have facilities for molecular diagnosis. However, it is of little help to prognosticate and assess the clinical severity of Bruck syndrome. Therefore, good clinical evaluation remains the key to delineate the basic abnormalities associated with this disorder.

In addition to multiple bone fractures and congenital joint contractures, patients with Bruck syndrome commonly have short stature, Wormian bones in the skull, clubfoot, and kyphoscoliosis. They mostly have white sclera as in the patient described herein, and normal hearing and vision. Individual case reports [14–18] have described additional dysmorphic features, as reported in this child. The clinical outcomes and prognosis have been variable in reported cases, with some reported children dying soon after birth [19] while other patients have reportedly lived up to the third [20] or fourth [21] decade in life.

The molecular defect underlying Bruck syndrome is a deficiency of bone-specific telopeptide lysyl hydroxylase, which results in aberrant crosslinking of bone collagen [22]. Lysine residues within the telopeptides of type I collagen in bone are underhydroxylated, leading to aberrant crosslinking. In contrast to bone, cartilage and ligament show unaltered telopeptide hydroxylation in Bruck syndrome, as evidenced by normal patterns of crosslinking. These underlying biochemical derangements give rise to predominant changes in bone that include congenital fractures and contractures, and increased fragility. Brenner *et al.* [23] performed electron microscopy of a bone specimen of a patient with Bruck syndrome and found osteoblasts with swollen mitochondria and dilated endoplasmic reticulum. A decrease in the diameter of the collagen fibrils was also noted, along with low mineral content and increased pepsin extraction of collagen 1.

It is important to rule out osteogenesis imperfecta in newborns with arthrogryposis multiplex congenita. Radiological imaging should therefore be performed early so that the affected child can be better looked after by measures such as padding of beds and gentle handling to prevent further fractures [19]. The mainstay of pharmacological management of Bruck syndrome is bisphosphonate infusions and vitamin supplementation, and the condition is treated similarly to most children with osteogenesis imperfecta [24]. However, prognosis is worse in children with Bruck syndrome given its association with joint contractures [25].

The disease progresses relentlessly in all patients, leading to complications such as severe limb deformities, short stature, progressive kyphoscoliosis, and multiple fractures [3]. Most children with Bruck syndrome treated with pamidronate had good to fair response. Therefore, pamidronate therapy with the same doses used in osteogenesis imperfecta is currently recommended for these patients. Early orthopedic consultation would be routinely required for most of these patients to help them in rehabilitation and recovery.

Arthrogryposis can be picked up antenatally on ultrasound from the second trimester onward. Severe variants of Bruck syndrome diagnosed during the antenatal period can be offered genetic counseling and the option of termination of pregnancy [26].

## Conclusion

The association of arthrogryposis with osteogenesis imperfecta is extremely rare and known as Bruck syndrome. Early diagnosis during the antenatal period is helpful in genetic counseling, assessment of severity, and exploration of the option of therapeutic termination.

## Data Availability

The datasets used and/or analyzed during the current study are available from the corresponding author on reasonable request

## Abbreviation

AMC: Arthrogryposis multiplex congenita
